# The conformational changes of Zika virus methyltransferase upon converting SAM to SAH

**DOI:** 10.18632/oncotarget.14780

**Published:** 2017-01-21

**Authors:** Han Zhou, Fenghua Wang, Haofeng Wang, Cheng Chen, Tianqing Zhang, Xu Han, Deping Wang, Chen Chen, Chen Wu, Wei Xie, Zefang Wang, Lei Zhang, Lanfeng Wang, Haitao Yang

**Affiliations:** ^1^ School of Life Sciences, Tianjin University, Tianjin 300072, China; ^2^ Tianjin International Joint Academy of Biotechnology and Medicine, Tianjin 300457, China; ^3^ Key Laboratory of Molecular Virology and Immunology, Institute Pasteur of Shanghai, Chinese Academy of Sciences, Shanghai 200031, China

**Keywords:** Zika virus, methyltransferase, crystal structure, SAH, antiviral drug development

## Abstract

An outbreak of Zika virus (ZIKV) infection has been reported in South and Central America and the Caribbean. Neonatal microcephaly potentially associated with ZIKV infection has already caused a public health emergency of international concern. Currently, there are no clinically effective vaccines or antiviral drugs available to treat ZIKV infection. The methyltransferase domain (MTase) of ZIKV nonstructural protein 5 (NS5) can sequentially methylate guanine N-7 and ribose 2′-O to form ^m7N^GpppA^2′Om^ cap structure in the new RNA transcripts. This methylation step is crucial for ZIKV replication cycle and evading the host immune system, making it a target for drug design. Here, we present the 1.76 Å crystal structure of ZIKV MTase in complex with the byproduct SAH, providing insight into the elegant methylation process, which will benefit the following antiviral drug development.

## INTRODUCTION

Zika virus (ZIKV), a member of the *Flavivirus* genus in the family *Flaviviridae*, is transmitted primarily by Aedes mosquitoes. It has attracted the attention worldwide due to the severe threats to public health [1–6]. ZIKV infection during pregnancy may cause microcephaly in newborn infants [3–7] and is also considered as a trigger of Guillain-Barré syndrome [7, 8]. ZIKV is closely related to Dengue Virus, West Nile Virus, and other flaviviruses. There are no clinically effective vaccines or antiviral drugs available to treat ZIKV infection to this date.

**Figure 1:**
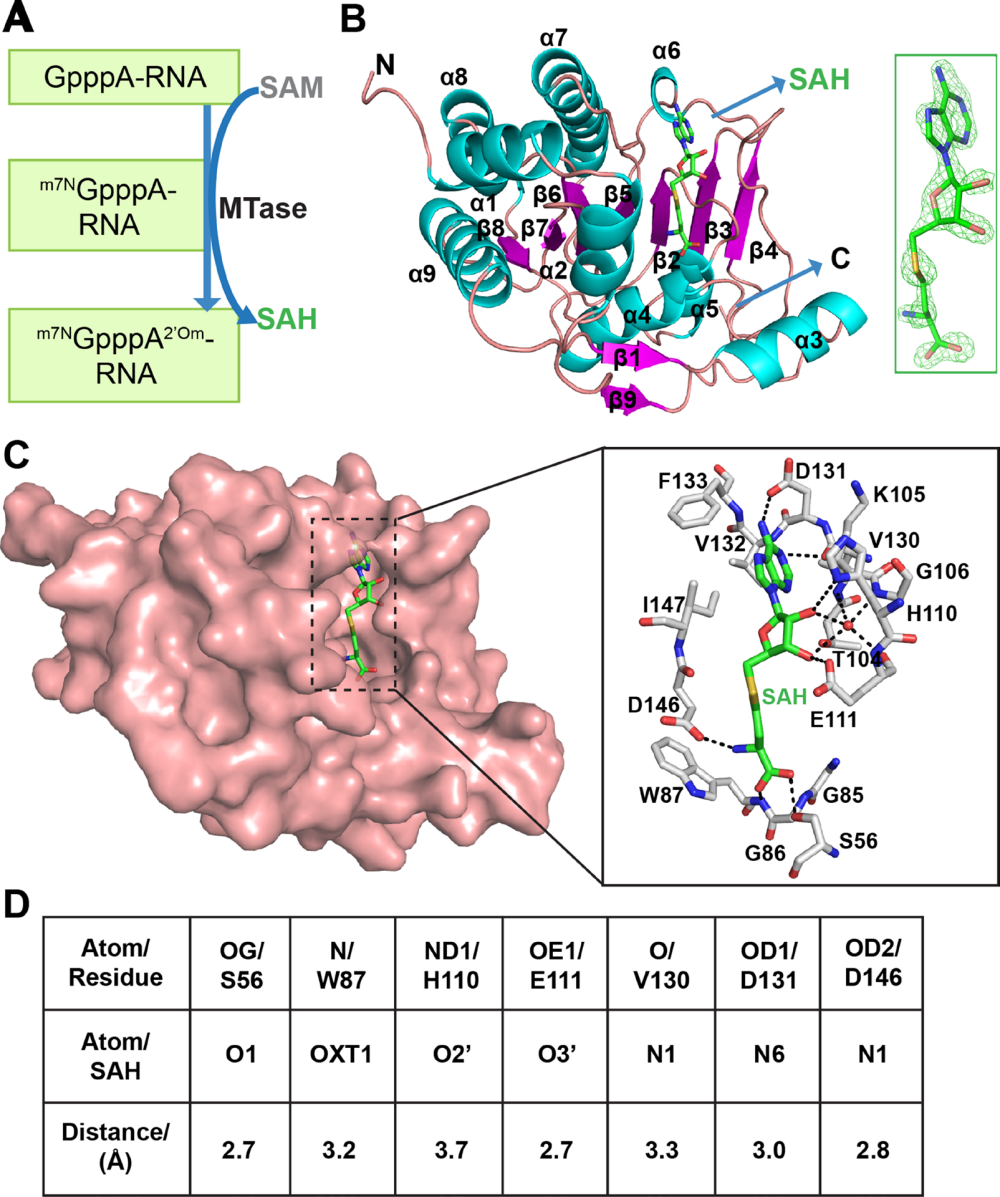
The structure of ZIKV MTase in complex with the byproduct SAH (**A**) The steps in which MTase sequentially methylates guanine N-7 and ribose 2′-O to form the ^m7N^GpppA^2′Om^ RNA cap structure. (**B**) Ribbon representation of the overall structure of MTase in complex with SAH (Green), which was clearly defined by the omit electron density map contoured at 3.0 σ. Cyan, helix; Magenta, strand; Salmon, loop. (**C**) The detailed interactions between SAH and MTase. (**D**) The distances are indicated for the hydrogen bonds in panel C.

ZIKV nonstructural protein 5(NS5) is a multi-domain protein, which has an N-terminal methyltransferase domain (MTase) and a C-terminal RNA dependent RNA polymerase (RDRP) domain. The MTase can sequentially methylate guanine N-7 and ribose 2′-O to form ^m7N^GpppA^2′Om^ cap structure in the new RNA transcripts [9–11]. The methylation starts with transferring the methyl group from the donor S-adenosylmethionine (SAM) to the methyl acceptor and then leaves the S-adenosylhomocysteine (SAH) as a byproduct (Figure 1A). This methylation step is crucial for ZIKV replication cycle and evading the host immune system [12, 13]. Defects in MTase are lethal for flaviviruses, which makes MTase a potential target for rational drug design against flaviviruses. Although the crystal structure of ZIKV MTase bound to SAM and 7-methyl guanosine diphosphate (7-MeGpp) was recently published [14], the stepwise of catalytic mechanism is still largely unknown. Here, we present the 1.76 Å crystal structure of ZIKV MTase in complex with the byproduct SAH, providing insight into the elegant methylation process, which will benefit the following antiviral drug development.

## RESULTS

### The structure of ZIKV MTase in complex with SAH

The crystal structure of ZIKV MTase was determined at 1.76 Å by molecular replacement using the structure of PDB ID: 5KQR [14] as the original search model with the *R*_free_ of 19.9% and *R*_work_ of 17.2% (Supplementary Table 1). The MTase forms a well-defined 3D structure consisting of nine α-helixes (cyan, α1-α9), nine β-strands (magenta, β1-β9), and multiple loop regions. Together, the α4-α8, β2-β8, and interspersed loops form the Rossman fold, which is a protein structural motif that binds nucleotides (Figure 1B, Supplementary Figure 1). In this structure, the byproduct SAH binds in the pocket of the N-terminal portion of the Rossman fold, which is primarily surrounded by α4-α6, β2, β3, β5, and adjacent loop regions. Clear electron density can be identified for SAH (Figure 1B and Supplementary Figure 2). In the complex structure, SAH is stabilized by hydrogen bond network formed between the ligand and the adjacent residues. Among them, H110, E111, V130, and D131 form the hydrogen bonds with the adenosyl group of SAH; S56, W87, and D146 form the hydrogen bonds with the homocysteine group of SAH (Figure 1C–1D). The ligand comes in contact with residues K105, G106, and E111 by ionic interactions. Furthermore, the side chains of F133, V132, and I147 can stack together with the base of SAH to enhance hydrophobic interactions (Figure 1C).

### The conformational changes of ZIKV MTase upon converting SAM to SAH

**Figure 2:**
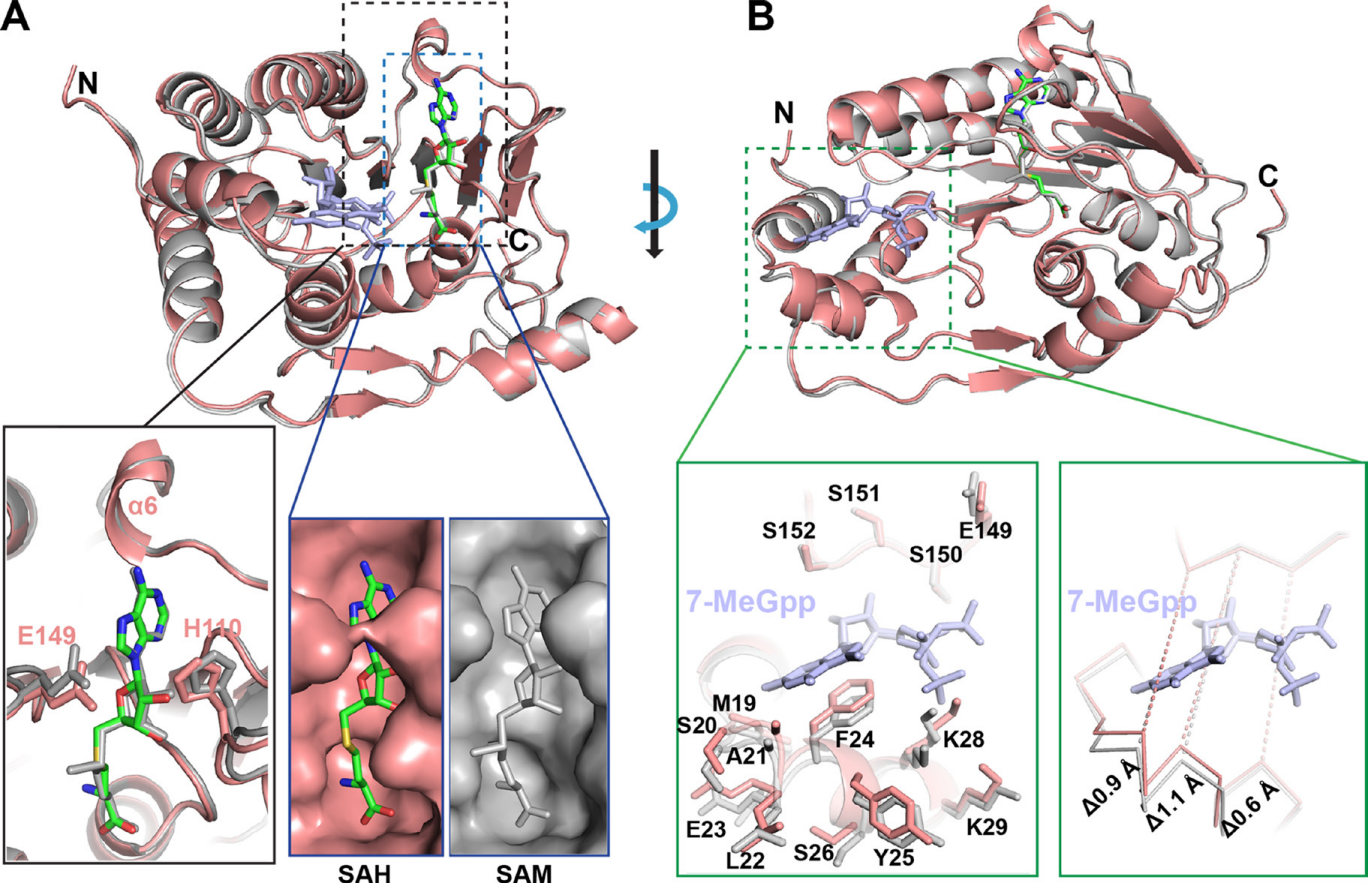
The conformational changes of ZIKV MTase upon converting SAM to SAH (**A**) Comparison of the conformational change of the binding pocket of SAM with that of SAH. (**B**) Shrinking 7-MeGpp binding channel in the absence of 7-MeGpp in comparison with the one in the presence of 7-MeGpp. (Salmon, our structure; gray, PDB ID: 5KQS).

In order to understand the stepwise catalytic mechanism for ZIKV MTase, we compared our structure with the published MTase structure in the presence of 7-MeGpp and SAM (Figure 2A). The overall structure of ZIKV MTase in complex with SAH is very similar with the reported one (PDB ID: 5KQS) with a RMSD of 0.242 Å (205 out of 266 Cαs). Meanwhile, the byproduct (SAH) was overlaid quite well with the substrate (SAM). However, we found that the size of the substrate/byproduct-binding pocket decreases dramatically upon converting SAM to SAH. The binding pocket was closed mainly through two gatekeeper residues: E149 and H110. And the surface area of the binding pocket decreased from 907 Å^2^ to 867 Å^2^. Additionally, the top region of the binding pocket forms a defined α helix (α6) instead of a random coil.

Interestingly, we noticed that the 7-MeGpp binding channel (Figure 2B) is significantly narrower in the presence than in the absence of 7-MeGpp. No obvious conformational changes were observed for the top region of the binding pocket, which is composed of the residues E149, S150, S151, and S152. However, the bottom region, which comprises residues M19, S20, A21, L22, E23 in the loop connecting α1 and α2, and residues F24, Y25, S26, K28, and K29 in α2 helix have significant conformational changes in both the backbone and the side chains. The main chain moved as far as 1.1 Å and caused the binding channel to shrink. Additionally, the residues ranging from 21 to 29 form a well-define α helix (α2) in our structure instead of a random coil in the published structure with 7-MeGpp (PDB ID: 5KQS).

Taken together, both the SAM/SAH binding pocket and the 7-MeGpp binding channel decreased in size upon converting SAM to SAH during catalysis.

## DISCUSSION

ZIKV MTase structure is highly conserved in the genus *Flavivirus*. The comparison of our structure and those of other flavivirus MTases has shown that this protein is well conserved among the genus. The RMSD between the structure of ZIKV MTase and those of Dengue virus (for 230 Cαs, PDB ID: 4V0Q [15]), yellow fever virus (for 227 Cαs, PDB ID: 3EVA [16]) and West Nile virus (for 228 Cαs, PDB ID: 2OY0 [17]) are 0.52 Å, 0.47 Å and 0.53 Å, respectively. The high homology of flavivirus MTases suggests that it could be a drug target to develop wide-spectrum antivirals.

The conformational changes of both SAM and 7-MeGpp binding sites of MTase upon converting SAM to SAH during catalysis has not been reported for any flavivirus. In our study, the comparison of these structures at high resolution provides accurate details to elaborate the stepwise catalytic mechanism for flavivirus MTase, providing the structural basis for rational drug design against ZIKV or other flaviviruses. The future research will focus on the structures of MTase in complex with initial substrate GpppA-RNA, final product ^m7N^GpppA^2′Om^-RNA, and other transition state mimics to elucidate the RNA capping mechanisms, which is essential for the viral replication cycle and evading host immune system.

## MATERIALS AND METHODS

### Protein expression and purification

The ZIKV MTase (residues 1–266) coding fragment was inserted to the BamHI and XholI restriction sites of the pET28b-SUMO vector for expression in the *Escherichia coli* BL21 (DE3). Cells were grown in LB medium at 37°C and then induced by 0.5 mM isopropyl-β-D-thiogalactopyranoside at 16°C. The cells were harvested and resuspended in lysis buffer A (25 mM Tris-HCl, pH 8.0, 0.5 M NaCl, 5% glycerol and 2 mM β-mercaptoethanol) and lysed by high pressure homogenization. The supernatant after centrifugation was loaded onto the Ni-NTA column (GE). The column was washed using buffer A supplemented with 20 mM imidazole and eluted using buffer A supplemented with 250 mM imidazole. After concentration by ultrafiltration, protein sample was diluted into buffer B (50 mM HEPES, pH7.5, 0.5 M NaCl, 5% glycerol), and the fraction containing (His)_6_-SUMO-ZIKV MTase was cleaved with ULP protease at 4°C overnight. The protein sample was then re-loaded on the Ni-NTA column to remove the (His)_6_-SUMO and the ULP protease and further purified by sequential chromatography: cation exchange column (Hi Trap SP 5 ml, GE) and gel-filtration column (Superdex 75 10/300 GL, GE). MTase was finally concentrated to 10 mg/ml (0.33 mM) and mixed with 1.98 mM SAH prior to crystallization.

### Crystallization, data collection, and structure determination

The crystals of ZIKV MTase were grown in the buffer (0.1M sodium citrate tribasic dihydrate pH 5.5, 22% (w/v) polyethylene glycol 1000) at 18°C using the microbatch-under-oil method. The crystals were cryo-protected by Parabar 10312 (previously known as Paratone oil). Diffraction data were collected at 100K at the Shanghai Synchrotron Radiation Facility (SSRF) beamline BL18U at a wavelength of 0.97776 Å. Diffraction data were processed using *HKL3000* [18]. The structure was solved using *PHENIX* [19] software package by molecular replacement using the structure of PDB ID: 5KQR [14] as the original search model. Multiple rounds of model building in *COOT* [20]and refinement in *PHENIX* were performed and led to a final model with *R*_work_ of 17.2% and R_free_ of 19.9%. The Ramachandran plot generated by PHENIX software package showed that the residues located in the favored and allowed regions are 98.1% and 1.9% respectively. No residues are located in the disallowed regions. The data collection and refinement statistics are summarized in Supplementary Table 1.

### Structural analysis and illustrations

*COOT* and *PYMOL* (The PYMOL Molecular Graphics System, Version 1.8 Schrödinger, LLC) were used for the structural analysis and illustration.

### Protein structure accession number

The refined coordinates have been deposited in the PDB under accession number 5WXB.

## SUPPLEMENTARY MATERIALS FIGURES AND TABLES


