# Supplementary material for: The conformational changes of Zika virus methyltransferase upon converting SAM to SAH
Source: Oncotarget. 2017 Jan 21;8(9):14830–4. doi: 10.18632/oncotarget.14780 (PMC5362447; doi:10.18632/oncotarget.14780)
Supplement: Supplementary file 1 [file oncotarget-08-14830-s001.pdf]

The conformational changes of Zika virus methyltransferase upon converting SAM to SAH

Supplementary Materials

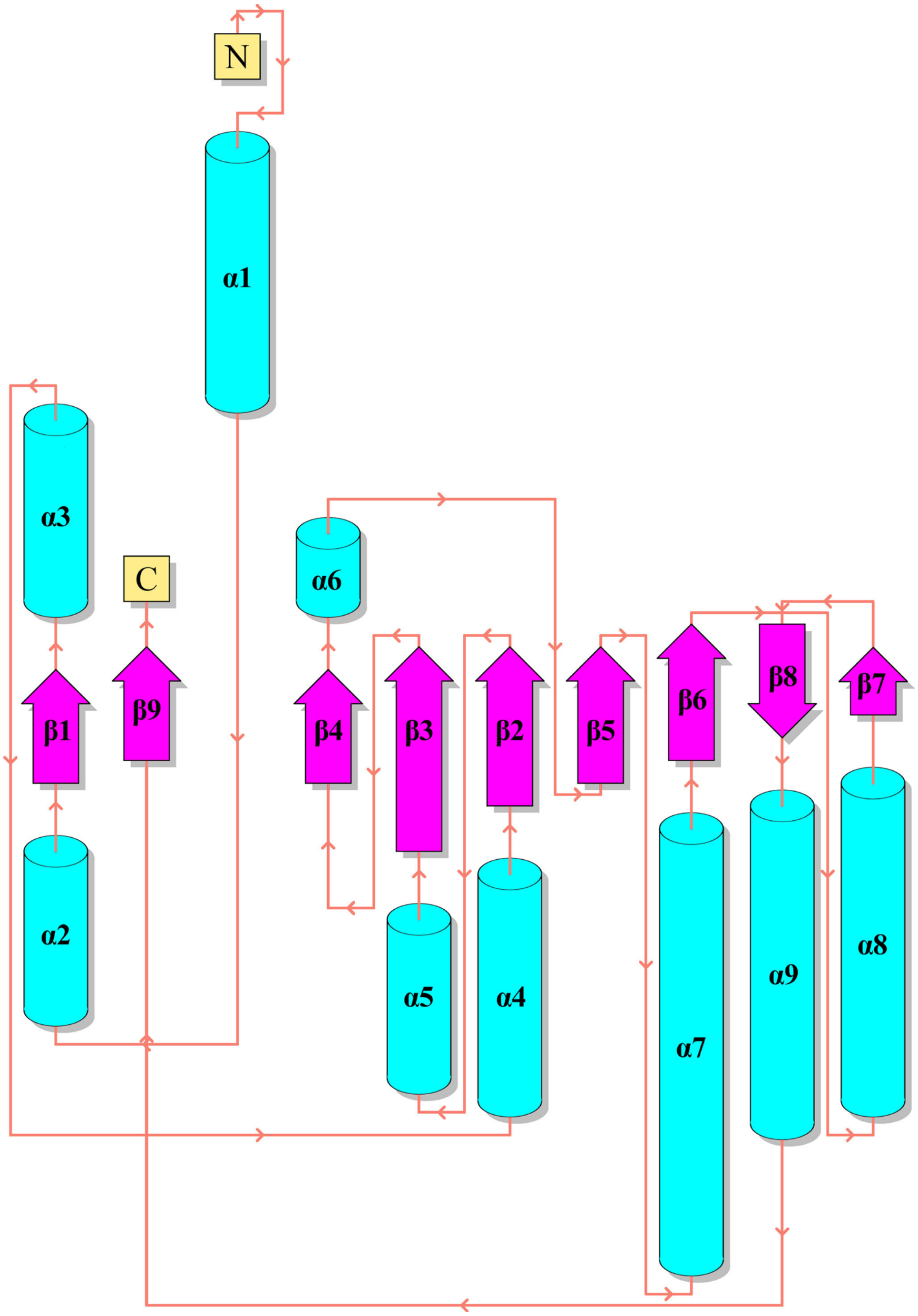

Supplementary Figure 1: The topology diagram of the protein fold of MTase. Color codes are the same as in Figure 1B

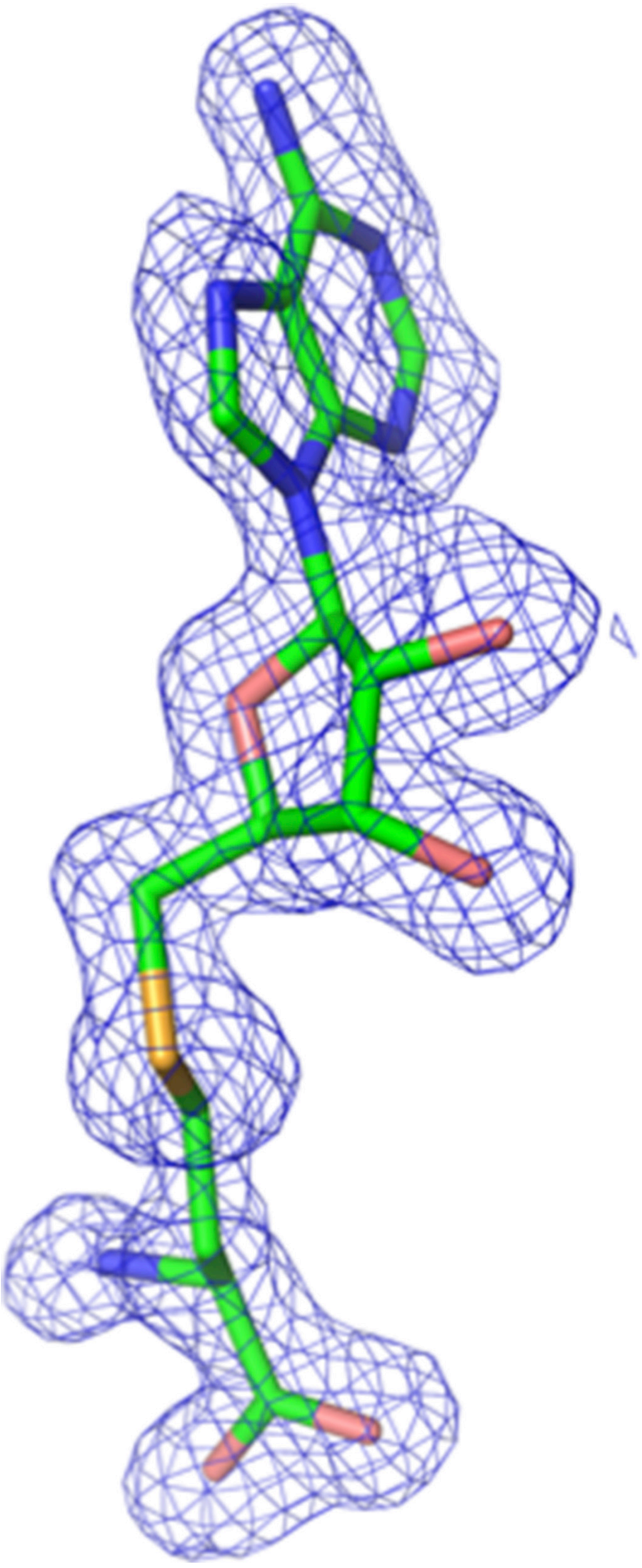

Supplementary Figure 2: Electron density maps of SAH. 2mFo-DFc map of SAH (blue) contoured at 1.2 σ.

Supplementary Table 1: Data collection and refinement statistics

	**MTase-SAH complex (PDB:5WXB)**
**Data Collection**	
X-ray Source	SSRF BL18U
Wavelength (Å)	0.97776
Space group	$P22_12_1$
Unit cell a, b, c (Å)α, β, γ (°)	38.0, 76.9, 109.6, 90.0, 90.0, 90.0
Resolution range* (Å)	50.0–1.76 (1.79–1.76)*
Unique reflections	32,293 (1,405)
Completeness (%)	98.8 (88.1)
Redundancy	4.9 (3.4)
$I/\sigma(I)$	15.6 (1.6)
*R*merge (%)	10.8 (50.2)
Refinement	
Resolution range (Å)	44.62–1.76 (1.83–1.76)
Reflections used for R_{free}	1,607 (132)
R_{work} (%)	17.2 (21.7)
R_{free} (%)	19.9 (24.0)
Number of atoms	2,407
Protein	2,048
Ligand	26
Water	333
Average B-factors (Å^2)	27.6
Protein	26.3
Ligand	18.5
Water	35.5
r.m.s. deviations	
Bond lengths (Å)	0.016
Bond angles (°)	0.82
Ramachandran plot (%)	
Favored	98.1
Allowed	1.9
Disallowed	0.0

*Numbers in the brackets are for the highest resolution shell.
